# Sick Leaves Pattern in a Tertiary Healthcare Facility in Saudi Arabia

**DOI:** 10.7759/cureus.11543

**Published:** 2020-11-18

**Authors:** Kossay Elabd, Abdullah Alkhenizan, Abdullah Aldughaither

**Affiliations:** 1 Family Medicine, King Faisal Specialist Hospital & Research Centre, Riyadh, SAU; 2 Family Medicine and Polyclinics, King Faisal Specialist Hospital & Research Centre-Alfaisal University, Riyadh, SAU; 3 Pediatrics, King Faisal Specialist Hospital & Research Center, Riyadh, SAU

**Keywords:** sick leaves, frequency, employees, healthcare organisation

## Abstract

Background

Frequent sick leaves in any healthcare organization is a critical problem that can undermine the patients' care through increasing the workload on other co-workers and costing the organization a lot of money.

Methods

This is a quantitative cross-sectional study looking at the frequency of sick leaves among employees of a large, tertiary healthcare facility in Riyadh. We randomly selected 474 employees, who were seen in family medicine clinics during a one-year period. We collected all the data retrospectively from their electronic medical records. Then we reviewed and analyzed all the data using SPSS software version 26.0 (IBM Corp., Armonk, NY).

Results

There was no difference in the sick leaves rate between males and females (p-value = 0.8618), but we saw a higher rate among younger employees (40 years old or less) compared to those 41 years or older (p-value <0.0001). We also investigated those who took four sick leaves or more during the period of the study, and we found that majority of them were nursing staff (31.71%), hospital assistances (24.39%) and housekeepers (14.63%). The commonest cause for taking sick leave in our study was viral upper respiratory tract infection (VURTI). Therefore, we studied the effect of influenza vaccine on the frequency of sick leaves and we found that those who took the vaccine were less likely to take a leave because of flu (p-value <0.0001, odds ratio 0.4067 with 95% CI: 0.2739-0.608).

Conclusion

Younger employees, nurses, hospital assistants and housekeepers are more likely to take sick leaves. Flu is the leading cause of sick leaves and influenza vaccine seemed to reduce its rate. In this study, we also discussed different methods that can be used by any healthcare organization to reduce the absence rate. Further studies are required to better manage the issue of excessive sick leaves.

## Introduction

Sick leave is a period of time that a worker is allowed to be away from work when he or she is ill, while absenteeism is a voluntary absence and usually for no good reason [1]. Absenteeism in the health sector is a long-standing problem that undermines healthcare delivery in many countries. Frequent unexpected and/ or prolonged absence from work can have many detrimental effects on patient care and the other healthcare workers remaining on duty. The other workers have to overwork to compensate, which can increase their stress level and can potentially increase the risk of mistakes. Moreover, it can cost the health organizations and the country a lot of money. In countries like the United States, it has been estimated that workers' illnesses and injuries are costing U.S. employers $225.8 billion annually [2].

Despite that, having a fair sick leave policy is essential for any employer, including those involved in healthcare, to allow employees when they are sick and unable to perform their duties to stay at home and to prevent the spread of infections to other co-workers. In the Kingdom of Saudi Arabia, many employers' sick leaves policies follow the Saudi labor law, which, states "The employee may be granted sick leave with full pay for a maximum of thirty calendar days and sick leave with seventy-five percent of full pay for sixty calendar days and sick leave without pay for thirty calendar days during any service year"[3]. Many employers made obtaining a medical certificate an essential requirement before granting the employee a sick leave with full pay. Although, some have waived the requirement for a medical certificate where the absence is for one day. Therefore, many employees, when they fall ill or need to take the day off for any other reason, they visit primary care clinics trying and faking symptoms to get a medical certificate, so they get paid for that day. This leads to an unnecessary increase in the waiting time of clinics for those who genuinely need medical care.

In our hospital and over the last few years, we have noticed an increase in the number of employees who are visiting family medicine clinics requesting medical certificates for sick leaves. Therefore, it was essential first to establish that we have a problem with frequent sick leaves requests and then to study this issue carefully and try to find a better way for dealing with absenteeism and the frequent unnecessary sick leave requests. This study aims to identify the pattern of sick leaves among employees from different hospital departments. For example, the demographics of those taking many sick leaves and finding out from which departments they are. We tried to quantify the issue with sick leaves and compare it to other local, national, and international healthcare organizations. We also tried to identify the leading causes for the sick leaves and if the influenza vaccine can help in reducing the frequency of these leaves.

This article was published as a pre-print (https://www.researchsquare.com/article/rs-33630/v1).

## Materials and methods

This paper is reporting a non-interventional quantitative cross-sectional study of the sick leaves pattern of the employees in King Faisal Specialist Hospital & Research Center (KFSHRC). The hospital is large tertiary healthcare based in the capital city of Saudi Arabia, Riyadh. We calculated the sample size using a 95% confidence level, 4.42% confidence interval (margin of error), and 50% population proportion while keeping in mind the hospital currently has 12,500 employees. The calculated sample size was 473. We randomly selected 500 employees using their medical record number (MRN). All the employees in this sample were seen in family medicine clinics between the beginning of January 2018 and the end of December 2018. We excluded 26 of them because of some missing data such as nationality or documented cause for their sick leaves. We collected the data from the 474 employees' electronic medical records. Then we statistically analyzed them using the SPSS software version 26.0 (IBM Corp., Armonk, NY). We reported descriptive statistics for continuous variables as mean with SD and summarized categorical variables as frequencies and percentages. We also tested the null hypothesis for continuous variables using t-test and ANOVA, while we used the Chi-square test to compare categorical variables. We set the level of statistical significance at p < 0.05.

## Results

Our sample of employees has an age range in years between 21 and 71. The mean age was 38.26 years (9.75 SD). Two hundred thirty-five (49.6%) of them were Saudis, and 239 (50.4%) were non-Saudi (see Table 1). Two hundred fifty-three of them (53.37%) were given at least one sick leave during 2018.

**Table 1:** Demographics of the study sample

Age	Mean 38.26 years (SD 9.75), range 21-75 years
Gender	Males (54%), females (46%)
Nationality	Saudi employees (235, 49.6%), non-Saudi employees (239, 50.4%)
Distribution by Non-Saudi	Philippines (119), India (40), Pakistan (10), Syria (8), Sudan (7), Egypt (7), USA (6), UK (6), Malaysia (6), others (30)
Distribution by top five departments	Nursing (21.94%), Administration (18.78%), Doctors “MD” (10.13%), Housekeepers (9.49%), Maintenance (8.65%)

Among those who took at least one sick leave, 136 (54%) were males and 117 (46%) were females. Nevertheless, there was no statistical difference between males and females (p= 0.4237). In addition, we found no statistical difference in the rate of sick leaves between local national and non-Saudi employees (p = 0.8904).

**Figure 1:**
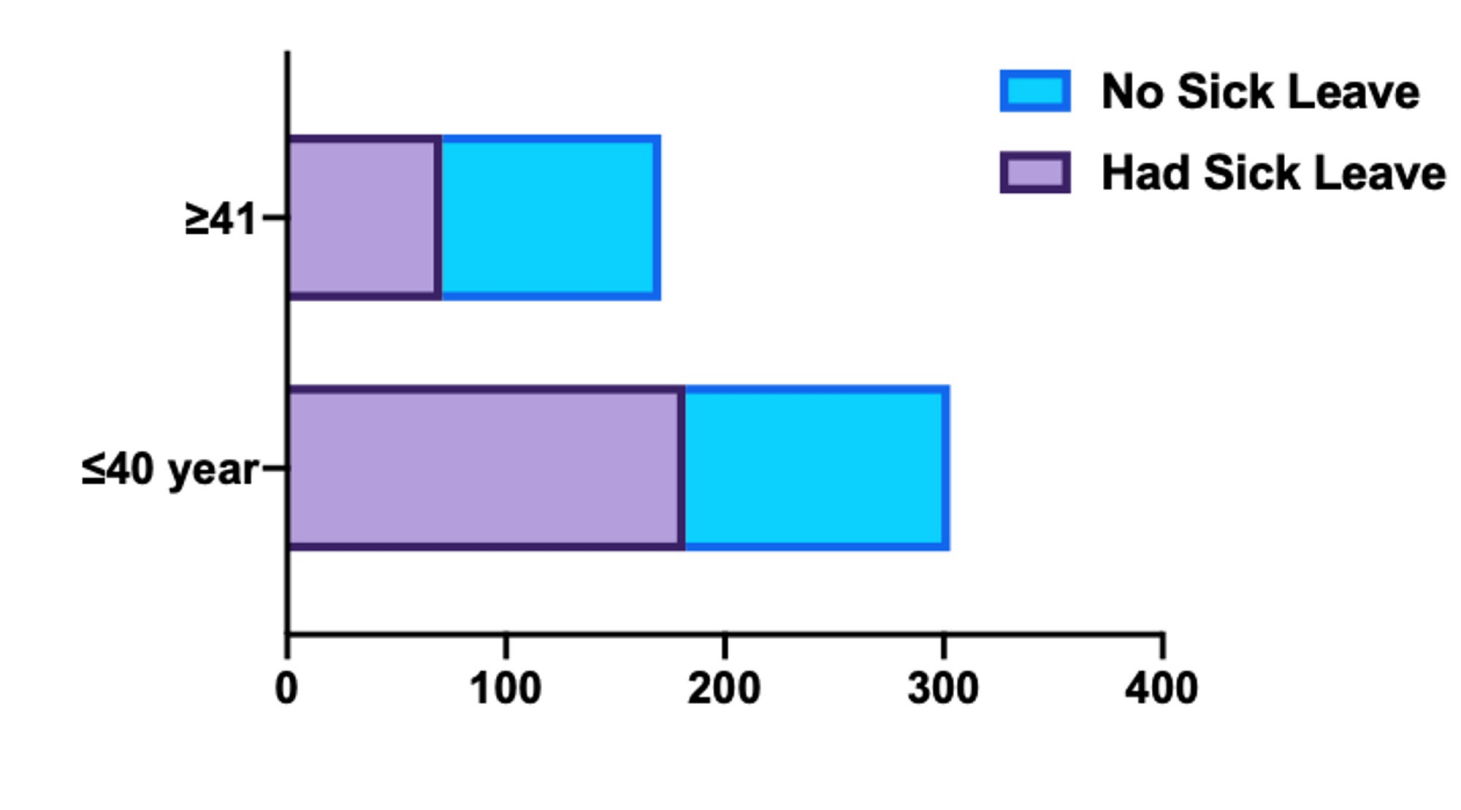
Percentages of sick leaves among different ages

We also calculated the percentages (out of the total sample: 474) for those who took and those who did not take sick leaves during 2018. We did this calculation for two groups, those who were 40 years or younger and those who were 41 years or older during 2018. Then we compared these percentages and found out that those who were ≤40 years had a statistically higher percentage (p <0.0001) (see Figure 1).

**Figure 2:**
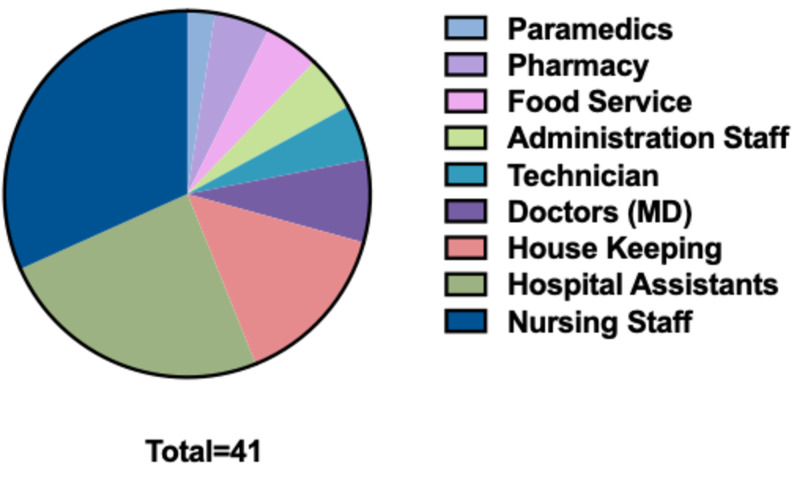
Distribution of those who took ≥4 sick leaves among different departments and professions

Most of those employees who took sick leaves had one to three leaves during the one-year study period, although some took as much as 13 sick leaves. We divided the employee sample into three categories, according to the frequency of their sick leaves. We found that 41 of them took four or more sick leaves. The rest took either no sick leaves (221 employees) or one to three sick leaves (212 employees). Among those who took four or more sick leaves, 23 (56%) were males, and 18 (44%) were females but no statistical difference (p-value = 0.4349). They were mainly nurses (31.71%), hospital assistants (24.39%), and housekeepers (14.63%) (see Figure 2).

**Figure 3:**
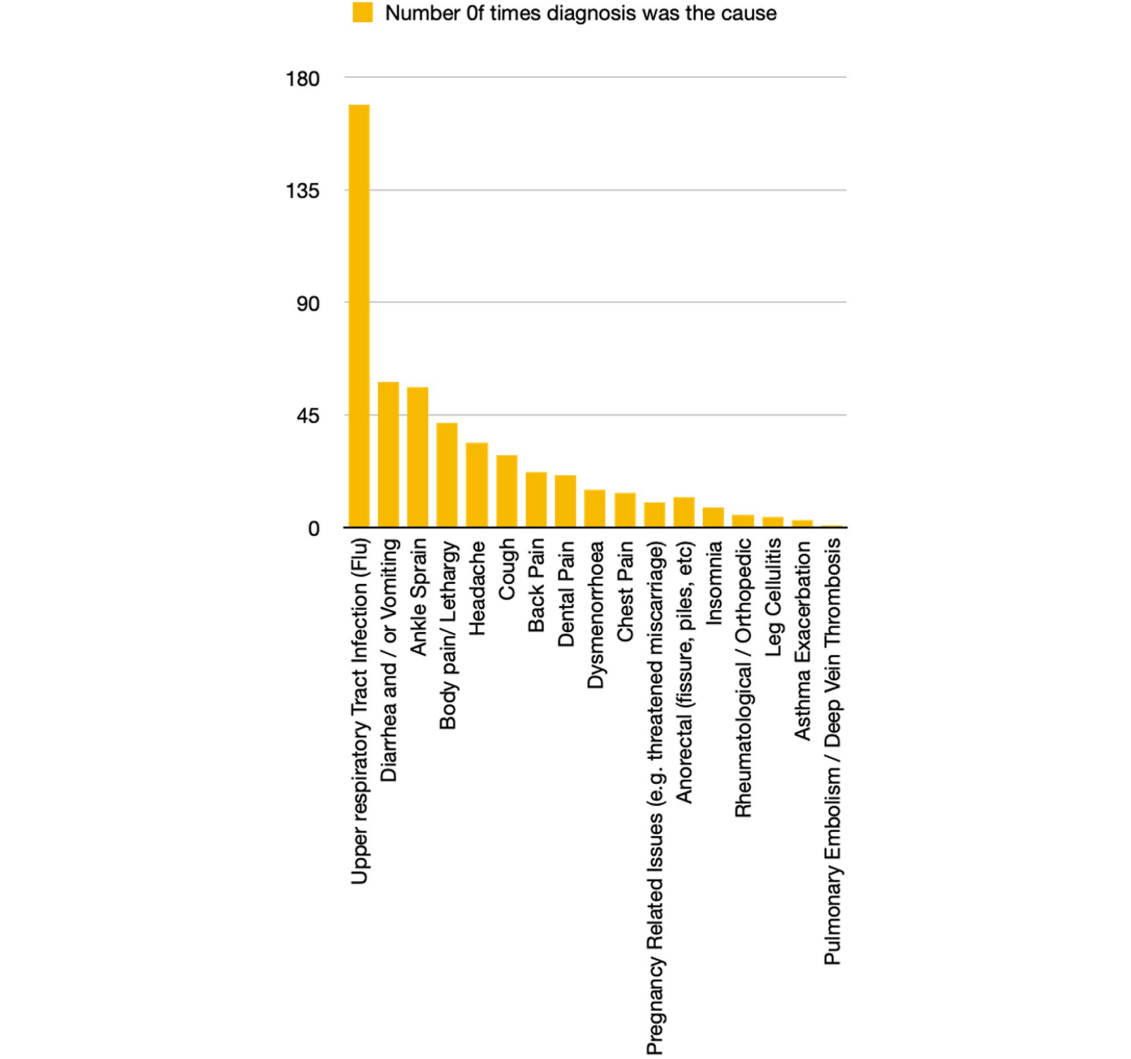
Causes of sick leaves

We identified a total of 503 sick leaves taken by the 253 employees who took sick leaves in 2018. We also identified 17 different diagnoses mentioned for these sick leaves. One hundred sixty-nine times (33.6%) were due to viral upper respiratory tract infection (VURTI or flu), which made it the most frequent cause (p <0.0001). Diarrhea and vomiting came second (58 cases or 11.53%) and ankle sprain came third with 56 cases (11.13%) (see Figure 3).

**Figure 4:**
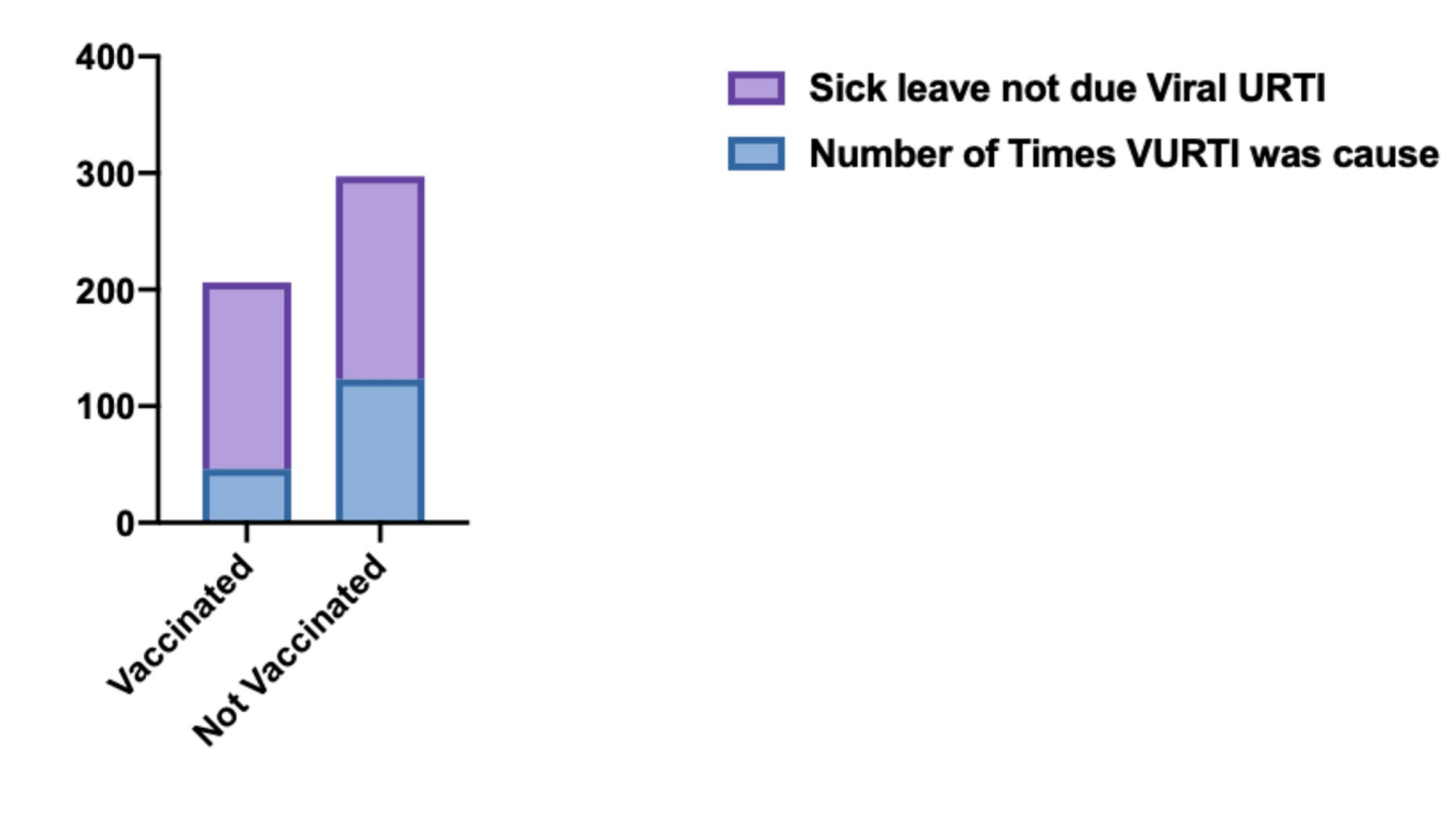
Flu as a cause of sick leave among vaccinated and those who were not vaccinated URTI, upper respiratory tract infection; VURTI, viral upper respiratory tract infection.

Since the highest number of sick leaves were taken for flu (or VURTIs), we investigated the effect of the influenza vaccine on the rate of sick leaves to see if those who took it were less likely to take sick leave. We did this in three stages: First, we compared the total number of sick leaves among those who were vaccinated and those who were not, and we did not find any statistical difference (p = 0.4583). Secondly, we divide the sample into five groups, those who did not take any sick leaves and those who took one, two, three, and those who took four or more sick leaves to find out if any of these groups had more influenza vaccine than the rest. We did not find any statistically significant difference (p= 0.5661). Finally, we looked at the employees who had any number of sick leaves due to flu or VURTIs. We calculated the relative frequency of sick leaves due to flu to the sick leaves due to other causes in both groups, vaccinated and no vaccine. Later we compared these relative frequencies in these two groups. We found the relative frequencies of flu to be less among vaccinated (p <0.0001, odd ratio 0.4067 with 95% CI: 0.2739-0.608) (see Table 2, Figure 4).

**Table 2:** Flu as a cause of sick leave among vaccinated and those who were not vaccinated URTI, upper respiratory tract infection; VURTI, viral upper respiratory tract infection.

	Number of times VURTI was the cause	No viral URTI (either no SL/ SL due to other cause)
Vaccinated	40	179
Not Vaccinated	88	224

**Figure 5:**
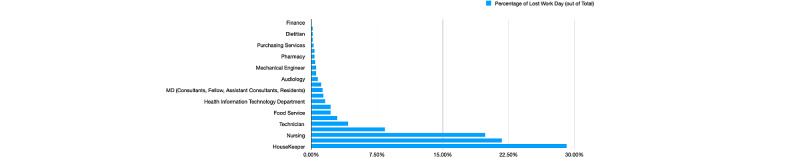
Distribution of the lost working days

The hospital has about 12,500 full-time employees. Just over a half (53.37%) of the 474 employees' sample who visited family medicine clinics had at least one sick leave during 2018. This sample lost a total of 1073 working days in one year, which means 4.24 days per absentee per year, and around 2.26 workdays per employee are being lost every year, and potentially a total of 28296 workdays are being lost every year in our hospital. We also found that housekeepers (312 days, 29.08%), hospital assistants (233 days, 21.71%), and nursing staff (213 days, 19.85%) were the highest contributors to these lost working days (p <0.0001) (see Figure 5).

## Discussion

We found our sick leaves rate to be 4.24 days per absentee per year and around 2.26 days per employee per year. If we compare these figures to other figures from other healthcare organizations in the Kingdom of Saudi Arabia, we can see our hospital had relatively higher rates of sick leaves. Two studies were both done in one hospital in Riyadh in two different years, 1993 [4] and 2009 [5]. The first showed a sickness absence rate of 1.69 days per absentee or 0.2 days per employee per year. On the other hand, our figures are relatively less than some international figures. In the United Kingdom, for example, the Office for National Statistics revealed that employees lost an average of 4.4 working days per employee in 2018 because of sickness or injuries [6]. Other European countries in 2016 had an average of 11.8 days per employee per year, with a range between 2.5 and 18.6 days/employee/year [7]. Also, in the United States, on average, employees take seven to nine sick leaves per year [8].

Employees who take an excessive number of sick leaves can cause several problems in the workplace. Whether these leaves were for real illness or not, they still have disruptive effects on patients’ care and on the other co-workers who had to work extra to compensate for their colleague's absence. In our study, we found that employees from key hospital departments were among the top requesters for sick leaves. They were nursing staff, hospital assistants, and housekeepers, consecutively. There is no clear consensus as to what might be considered as "Excessive Absenteeism." Some consider three days of sick leaves in 90 days as excessive [9]. Some others calculated the rate of the sick leaves for all the employees to know what rate can be regarded as a benchmark, taking into account the number of working days and the number of employees in the business [10]. We decided to choose four sick leaves per year or more as an indication that the employee is taking unusually more frequent sick leaves than usual, as from personal experience, most of the employees take between one and three sick leaves per year. In our sample, 253 employees took at least one sick leaves in the study period, and among them, 41 employees (16.2% of the absentees, or 8.6% of the sample employees) took four sick leaves or more in that year.

Most of the sick leaves were taken by relatively younger employees (≤40 years old) compared to older employees (≥41 years old) (p <0.0001). A similar finding that indicates younger employees are more likely to take sick leaves has been identified in many other previous studies in different countries [11,12]. This finding of younger employees having a higher rate of sick leaves is a novel finding, given the relatively low rate of chronic diseases among younger people [13], which potentially mean there should be other reasons apart from ill health that account for the increase in the rate of the sick leaves among this age group.

VURTI is the most common cause of sick leaves in our study (33.6%, p-value <0.0001). The same finding was revealed in many previous studies in Saudi Arabia [4,5,14] as well as the UK [6]. Therefore, we tried to find out if taking the influenza vaccine can help us in reducing the rate of sick leaves. Although we did not see any significant difference in the sick leaves rate between vaccinated and unvaccinated employees (p= 0.4583), we found those who were vaccinated had a significantly less relative frequency of sick leaves to due flu compared to those who were not vaccinated (p <0.0001, odd ratio 0.4067 with 95% CI: 0.2739-0.608). The efficacy of the influenza vaccine in reducing the sick leave rate has been confirmed in many studies [15,16].

Suspecting those who are requesting medical certificates for insincere reasons can be very challenging. Sometimes some employees request sick leaves for reasons other than being mentally or physically ill. Doctors have a significant role in reducing the number of sick leaves due to dishonest reasons. This can be achieved through a proper patient assessment and evaluation to uncover their ability to perform their duties while keeping in mind any risk of spreading infectious diseases if their employees go back to work.

Doctors have to be alert to recognize those who might be pretending to be ill, which, unfortunately, can be practically very difficult. For example, a patient claiming to have a headache or back pain when the doctor cannot prove otherwise. However, at least if in doubt, it may be beneficial to certify the patient with shorter sick leaves if it was medically justifiable. Some indicators have been suggested by some experts in staff management, which might help us identify those who might be wrongfully claiming illness to get a medical certificate for sick leave [17,18]. For example, a frequent sick leave requester, or whenever there is a pattern for the leaves, such as for the day(s) before or after weekends or holiday, or for days when there are heavy duties, for days which coincide with the declined annual leave days.

Some countries have tightened the regulations around having paid sick leaves in an attempt to reduce absenteeism. In the United States, for example, there are no federal legal requirements for paid sick leave [19]. Also, in some countries in eastern Europe, the sickness benefit levels and coverage have been reduced in an attempt to reduce sick leaves [20]. Although these measures might reduce the rate of sick leaves, but can potentially force involuntary presenteeism, which is defined as coming to work despite illness as a result of fear of losing the job [1]. Presenteeism has its risks of reducing work productivity and the risk of spreading infections. Therefore, having no policy for sick leaves in any healthcare organization is not advisable.

Many experts in staff management have suggested a few interesting measures to improve employees' job satisfaction and engagement and subsequently reducing unnecessary frequent sick leaves. For example, ensuring all employers have a clear paid sick leave policy and attendance policy [21]. But, at the same time, disciplinary actions should be clearly stated for those who frequently break the rules to avoid abusing these policies. Also, considering some allowance for non-medical leaves and flexible working arrangements such as part-time jobs or work from home for those who genuinely need it. These allowances are especially important for those who might have particular needs, such as those looking after young children, disabled or elderly people. Another noteworthy way is to make any employee on sick leave feel missed and appreciated, through, for example, sending him a "get-well-soon card" or a phone call, as this might increase his desire to go back to work and make him less likely to take time off in the future unless he really needs it. In addition, ensuring employees have job security as perceived job insecurity can potentially increase absenteeism. Feeling of job security can be achieved through excellent communication with employees to make them understand their duties and introduce any new changes that might happen in the organization well in advance to reduce any confusion or uncertainty among them and therefore improves their productivity and engagement [22].

Moreover, providing employees with proper training and new learning opportunities in the workplace can be another way of improving employees’ engagement and productivity and subsequently reducing their absence. Last but not least, looking after employees' health is an essential way of reducing sick leaves among employees and should not be forgotten. However, many studies that looked at the relationship between providing employees with essential healthcare and the rate of sick leaves and absenteeism have shown controversial results. For example, there are three studies that have been done in the United States and came up with relatively contradictory results; in the first study, they found that providing employees with healthcare insurance reduced the number of absenteeism [23]. While in the second study, there was no significant relationship, although the findings in this study were in old employees [24]. However, in the third study, they found having healthcare insurance is associated with less absenteeism, but for those employees who had a higher level of access to healthcare, the absenteeism rate was higher [23]. Employers should still be looking after their employees' health, at least through having appropriate health and safety policies: such as providing healthcare workers with the essential personal protective equipment (PPE) free of charge and through different other measures to ensure the workplace has a safe and healthy environment. Also, healthcare employers should provide their employees with the annual influenza vaccine as it reduces the incidence of flu or VURTIs [25].

All frequent sick leave requesters for no apparent major temporary illness should be referred to Occupational Health for the proper assessment, where they will be assessed to answer many critical relevant questions. For example, what is the underlying medical condition that is causing this absenteeism, estimation of how long the employee needs to be absent from work, and on returning to work, would he be able to resume his regular duties or would he need to have some modification to his duties or would he need to go into an early retirement [26]. At the same time, it is essential that all the physicians who are dealing with the absentee must be keeping in their mind that there might be other factors other than the employee's health, making him taking time off work. These factors could be things like employees’ health believes and sickness behavior, motivation to attend, job satisfaction, perceived physical and psychosocial work conditions, anticipated job demands, and work-related stress such as those due to conflicts [26].

Our study is the first study of its kind in our hospital, which looked at the pattern of sick leaves among the hospital employees in such details. We found many vital results which will help us deal with this issue in a better way. Nevertheless, this study had some limitations that are worth mentioning. Firstly, we only included hospital employees, although, in family medicine clinics, we see all the employees and their dependents. Many of the dependents have jobs outside the hospital, and many of them come to family medicine clinics for sick leaves. We feel it would have been more informative to see the pattern of sick leave among everybody who is attending these clinics. Secondly, we only looked at sick leaves and did not include those who were absent without sick leaves as it was not possible to recognize them from their medical records. Lastly, studying "presenteeism," which is attending to work while being ill, would have been an excellent addition to this study.

We need to consider further studies in the future to find out why there was a higher rate of sick leaves among younger employees and among particular key departments or professions within the hospital such as nursing staff, hospital assistants, and housekeepers. It is essential to investigate the actual reasons behind the higher rate of sick leaves among them, as this might help the hospital tackling the problem of absenteeism in the future.

Besides, the policymakers in the hospital might need to consider making flu vaccination mandatory to all hospital staff and introduce certain upper limits for the sick leaves duration for some of the medical diagnoses [27]. They might also need to consider introducing more measures, like those mentioned before, to reduce the number of sick leave abusers.

## Conclusions

We have a relatively higher rate of sick leaves compared to what has been reported in other hospitals in Saudi Arabia. On the other hand, we have less sick leaves rate than those reported in some other countries like some countries in Europe and the United States. Absenteeism in our hospital is happening at a higher rate amongst the employees who are 40 years old or younger, nurses, hospital assistants, and housekeepers. More studies are needed to explore these findings further to help us find a better way for reducing the incidence of absenteeism. Doctors need to be more vigilant in spotting sick leave abusers and try to give the minimum number of sick leave days whenever appropriate. Moreover, doctors who are giving the medical certificates for sick leaves, have to always keep in their mind that there might be other personal or psychological reasons other than a physical illness that is forcing the employee to frequently seek sick leaves. Besides, hospitals’ policymakers might need to consider introducing some measures to reduce absenteeism, such as making influenza vaccine mandatory for all employees, setting up an upper limit for sick leave days given to specific diagnoses, and try to make the workplace better and happier to improve employee's engagement and job satisfaction. Also, all frequent sick leave requesters who do not have a clear temporary medical reason should be referred to occupational health for fitness for work assessment. Finally, each healthcare organization should have a clear disciplinary policy for those who frequent sick leave abusers.
